# Nanocomposites of ZnS and poly-(dimethyl)-block-(phenyl)siloxane as a new high-refractive-index polymer media

**DOI:** 10.1186/1556-276X-7-181

**Published:** 2012-03-08

**Authors:** Natalia Sergienko, Dmitri Godovsky, Boris Zavin, Minjong Lee, Minjin Ko

**Affiliations:** 1 A.N.Nesmeyanov Institute of Organoelement Compounds of Russian Academy of Sciences (INEOS RAS), Vavilova St. 28, Moscow, 119991, Russia; 2 LG Technology Centre Moscow, Paveletskaya sq 2/3, Moscow, 115054, Russia; 3 LG Chem Research Park, Yuseong-gu, Daejeon, 305-380, Republic of Korea

**Keywords:** high-refractive-index media, polymer-nanocomposite, metallosiloxane

## Abstract

In the present paper, we describe a new and original method to obtain transparent, siloxane-based composites, with high refractive index (up to 1.68). The method is based on the decomposition of Zn-siloxane, mixed with a poly-(dimethyl)-block-(phenyl)siloxane matrix in different ratios. It was found that after treatment of such mixed metal-containing polymer blend with H_2_S, the nanoparticles of ZnS are formed, with the size in a 1- to 5-nm range, which allow effective increase of the refractive index of the nanocomposite mixture with poly-(dimethyl)-block-(phenyl)siloxane without loss of film transparency. We succeded to increase the refractive index from 1.54 (pure matrix) up to 1.68 (composite with a ZnS content of 4.6 vol.%). The siloxane-based compositions are optically transparent, which makes it possible to use them as light-emitting diodes or solar cell sealants or adhesives.

## Background

At present, a lot of efforts are concentrated on the synthesis of transparent polymer materials with high refractive index, which are used for different purposes: in light-emitting diode [LED] production, in photovoltaics, as light waveguides, and in other areas of material science and optics. The common polymers do not allow to produce a material with a refractive index of more than 1.54 to 1.55 [1]; thus, special methods are necessary in order to obtain the transparent polymer media with a refracti ve index higher than the numbers mentioned above.

One of the ways to obtain the polymer material, having the same properties as a polymer matrix but much higher refraction index, is to make the nanocomposites, introducing into the polymer matrix nanoparticles with the size much less than the wavelength of light, with much higher index of refraction. Traditionally, such materials as TiO_2 _and ZrO_2 _are used for these purposes [2-6], and mainly, water-soluble polymers are used. However, the main problem in making such nanocomposites is to obtain the homogeneous distribution of the nanoparticles in the polymer matrix, preventing their agglomeration, which causes extended optical scattering. The agglomeration of nanoparticles leads to high scattering and loss of optical transparency of the nanocomposites. One of the ways to prevent it is to modify the surface of the nanoparticles with organic ligands or oligomer chains [7], making capped nanoparticles which allow more or less homogeneous distribution of particles in the polymer. By this method, e.g., ZnS nanoparticles capped with thiophenol-4-thiomethyl styrene were introduced into ZnS-poly(urethane-methacrylate) macromer matrices, with concentrations up to 86 wt.% and an increasing refraction index from 1.63 to 1.78. The other method to make homogeneous distribution of nanoparticles is the *in situ *synthesis of nanoparticles from metal-containing polymers. Polyimides, containing TiO_2_, were synthesized using this method from Ti-imides [8], and the effective refractive index reached for such a nanocomposite was as high as 1.73. On the other hand, we found no references in the literature, to the research, where nanoparticles were obtained by means of metal-containing polymer decomposition in the case of siloxane-based compositions, even though the siloxane-based compositions with high refractive index, like those of Lu et al. [9], are of big practical interest as sealants, adhesives, and optical fillers for solar cells, light-emitting diodes, waveguides, lasers, and other optical applicatons.

In our paper, we would like to present a new and original method to obtain such nanocomposites, using the mixture of Zn-containing metallosiloxane with a poly-(dimethyl)-block-(phenyl)siloxane matrix. We developed the special method to obtain high-refractive-index nanocomposites, suitable for various optical needs. The sulfides and namely ZnS nanoparticles were obtained as a high-refractive-index filler even though it is much less common to use sulfides and not oxides as a high-refractive-index filler. Nevertheless, the refractive index of pure ZnS is as high as 2.37 [10], and the optical bandgap value is 3.2 eV, which basically makes it as much attractive filler material as e.g. TiO_2 _or ZrO_2. _At the same time, ZnS nanoparticles are characterized by much less amount of non-stoichiometry and defects and are inert even at high temperatures in oxygen-containing atmosphere.

## Methods

### Synthesis of poly(Zn-)phenylsiloxane

A three-necked flask, equipped with reflux condenser, dropping funnel, thermometer, and magnetic stirrer, was loaded with 25.94 g (0.108 mol) of phenyltriethoxysilane, C_6_H_5_Si(OC_2_H_5_)_3_, and 122 ml of C_2_H_5_OH (rectified, 96%), containing 3.99 g (0.216 mol) water. Metallic Na in an amount of 2.48 g (0.108 mol) was introduced in the reaction mixture step-by-step by small portions with stirring. After the dissolution of Na, the reaction mixture was heated to boiling, and a solution of 7.35 g (0.054 mol) ZnCl_2 _in 122 ml of absolute C_2_H_5_OH was introduced to the reaction flask through the funnel. After that, 200 ml of toluene was additionally charged to the flask.

The reaction mass was heated for 4 h at boiling point. After cooling, the precipitate of NaCl was removed by filtration. The filtrate was evaporated in a rotary evaporator under vacuum of a water jet pump. The resulting resin was dried in a boiling water bath (at a residual pressure of 1 mmHg.) to a constant weight, obtaining 18.28 g of white powder; the yield is 86%. The results of elemental analysis (in mass percent) found that Si = 15.2%, Zn = 16.7%, and Zn/Si ratio = 1:2.2, and the calculated values for C_12_H_10_Si_2_O_2_Zn are Si = 16.6%, Zn = 19.3% and Zn/Si ratio = 1:2.

### Preparation of Zn-containing compounds

The samples for investigations are prepared on the basis of a toluene solution of synthesized poly(Zn-)phenylsiloxane (concentration is 100 mg/ml) and poly-(dimethyl)-block-(phenyl)siloxane resin (concentration is 275 mg/ml). The poly-(dimethyl)-block-(phenyl)siloxane resin contains di- and trifunctional units (CH_3_)_2_SiO- and C_6_H_5_SiO_1.5_-. Polymer compositions were prepared by mixing solutions of siloxane resin and metallosiloxane in predetermined proportions. The curing agent K-18 in amounts of 10% and 20% by weight of dry polymer was added for the vulcanization. Prepared this way, the polymer composition was poured onto a smooth substrate and kept in air at 20°C to 25°C. The transparent, solid vulcanized films are formed due to interaction with air moisture during toluene evaporation. Characteristics of the compositions and their vulcanizates are presented in Table 1. Half of the prepared vulcanizates were further subjected to exposure to gaseous H_2_S for 24 h at 25°C ('sulfidization' process). Another part was used as a 'control' sample for comparison.

**Table 1:** Comparison of theoretical and experimental Zn contents as determined by AAS

	Zn (wt.%)		Si (wt.%)		Zn/Si ratio	
	Experiment	Theory	Experiment	Theory	Experiment	Theory
Sample 1	16.64; 16.82	-	15.10; 15.28		1.0:2.1	1.0:2.0
Average values	16.71	19.27	15.19	16.56		
Sample 2	18.10; 18.15	-	15.16; 15.26	-	1.0:1.96	1:2.0
Average values	18.13	19.27	15.21	16.56		

### Sulfidization of Zn-phenylsiloxane

Sulfidization by H_2_S was done according to the general scheme:

$${\left[{\text{C}}_{\text{12}}{\text{H}}_{\text{1}0}\text{S}{\text{i}}_{\text{2}}{\text{O}}_{\text{2}}\text{Zn}-\right]}_{\text{x}}+\;{\text{H}}_{\text{2}}\text{S }\to {\left[{\text{C}}_{\text{12}}{\text{H}}_{\text{1}0}\text{S}{\text{i}}_{\text{2}}{\left(\text{OH}\right)}_{\text{2}}\right]}_{\text{x}}+\text{ ZnS}\text{.}$$

The result is a nanosized particle of ZnS, uniformly distributed in the volume of the matrix of poly-(dimethyl)-block-(phenyl)siloxane resin.

Appearance of vulcanizates after sulfidization was virtually unchanged. The only exception are the samples with the highest zinc content which appear as slightly turbid, caused by scattering. At the final stage, all samples (sulfidized and control) were subjected to stepwise thermostating on the next regime: 50°C to 60°C (2 h) and then at 100°C to 110°C (2 h). The ratio between components of the final compositions (sulfidized and non-sulfidized) can be seen in Table 2.

**Table 2:** Formulation of compounds and vulcanizates

	Zn-phenylsiloxane			Organosiloxane resin + 10% K-18			Content of Zn (%)	ZnS (vol.%)
	%	g	ml	%	g	ml		
A1	0			100			0	0
A11S	20	0.069	0.69	80	0.275	1	3.6	1.39
A12S	30	0.118	1.18	70	0.275	1	5.4	2.13
A13S	40	0.18	1.8	60	0.275	1	7.3	2.95
A14S	50	0.14	1.4	50	0.137	0.5	9.1	3.76
A15S	60	0.12	1.2	40	0.08	0.28	10.9	4.61
A16S	70	0.14	1.4	30	0.06	0.22	12.7	5.51
A17S	80	0.16	1.6	20	0.04	0.14	14.5	6.44
A18S	90	0.18	1.8	10	0.02	0.08	16.3	7.42

### Preparation of films for optical measurements

Two kinds of films were made for optical measurements: self-supporting thick films (thickness 50 to 70 μm), which were made by dip casting in Petri caps, and thin films on silicon substrates, which were made for refraction index measurement.

The silicon (111) substrates were pretreated by usual RF-plasma in order to provide the proper surface quality. The films with the thickness from 70 to 500 nm were deposited by means of spin coating at a rotation speed in the range from 300 (500 nm) to 1,500 (70 nm) rpm. Thick films were mechanically durable and optically transparent. It is necessary to note that films with high ZnS content (A17S, A18S, A19S) were turbid and did not show optical transparency.

### Characterization of nanocomposites

**Figure 1:**
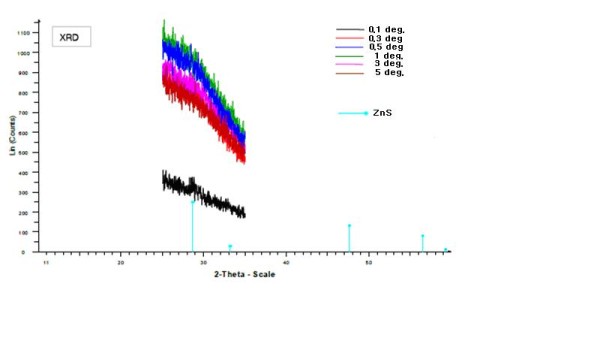
**XRD spectra of nanocomposite A14S taken under different incident angles ranging from 0.1 to 5**.

**Figure 2:**
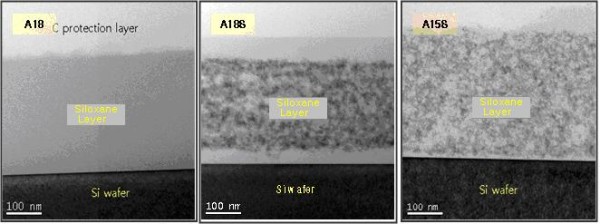
**TEM image of nanocomposites A15S-sulfidized (a), A18-non-sulfidized (b), and A18S-sulfidized (c)**.

**Figure 3:**
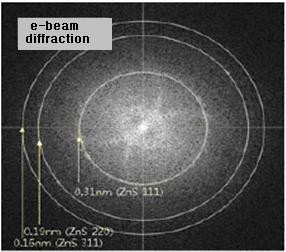
**HR-TEM image of nanocomposite A14S**.

X-ray diffraction [XRD] (Figure 1), transmission electron microscopy [TEM] (Figure 2) and high-resolution TEM [HR-TEM] (Figure 3) were performed for all nanocomposites, both sulfidized and non-sulfidized. The formation of ZnS nanoparticles after treatment with H_2_S was confirmed by TEM (Figure 2) and HR-TEM (Figure 3). The size of the particles, determined by TEM, was found to be 1 to 3 nm, slightly increasing with the increase of ZnS concentration. At the same time, TEM images of sulfidized samples (Figure 2) showed a clear nanodomain structure for some of the samples; the samples not treated with H_2_S did not show any nanoheterogeneity, and the samples with low ZnS content did not show any clear particles at TEM even though the refraction index changed drastically.

**Figure 4:**
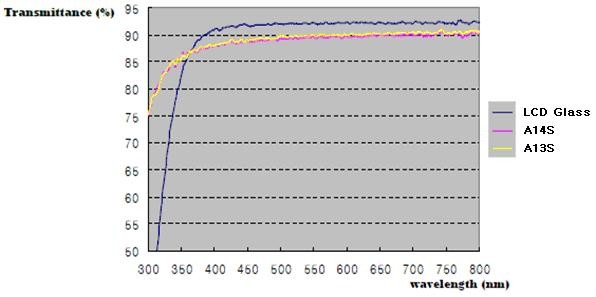
**Optical absorbance spectra**. Pure LCD glass (blue line), A13S (yellow line), and A14S (red line).

XRD spectra (Figure 1), taken for compositions with a whole range of concentrations, did not show a crystalline structure of the nanoparticles, but HR-TEM images of these samples resolved a crystalline structure - clearly seen as a face-centered cubic [FCC] lattice of ZnS (Figure 3). For the samples with higher loadings, there were still no XRD peaks associated with the FCC-type lattice of ZnS, but electron diffraction patterns can be clearly seen from HR-TEM images (Figure 4).

### Measurement of optical properties

**Figure 5:**
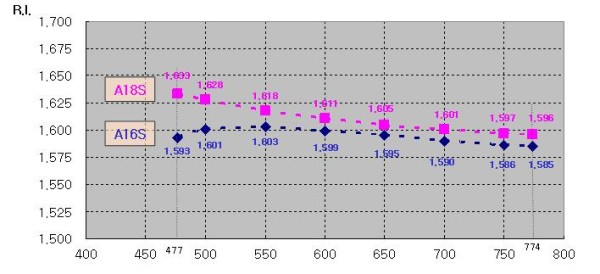
**Dependence of RI on the wavelength of composites for A18S and A16S**.

**Figure 6:**
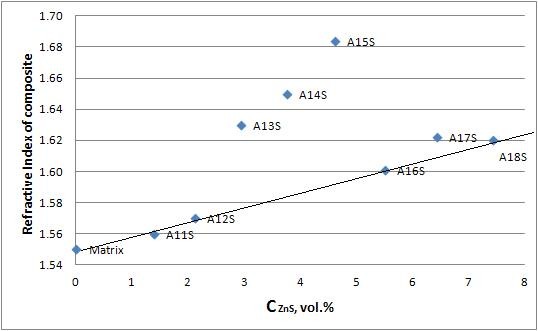
**RI dependence on ZnS content (vol.%), straight line - effective media approximation**.

Such optical properties as optical absorbance (Figure 4) and refraction coefficient (Figures 5 and 6) were measured in a broad range of ZnS concentrations and wavelengths. The absorbance spectra of samples, taken using a PerkinElmer 2500 spectrophotometer (Waltham, MA, USA) in the wavelength range from 300 to 800 nm, can be seen in Figure 4. They show the degree of transparency of the composite layer and the absence of significant scattering as well for the samples A12S and A13S. The absence of scattering could be observed by the eyes as well.

Refraction coefficient measurement was done using an ellipsometric refractometer in the wavelength range from 475 to 774 nm. The dependencies of the refraction coefficient on the wavelength of the samples can be seen in Figure 5. Also, the dependence of the refraction coefficient on the ZnS concentration was made and can be seen in Figure 6.

It is necessary to note here that it was impossible sometimes to correctly measure the refractive indices of the samples with high ZnS loading, starting from A16S, due to the fact that agglomeration of nanoparticles leads to non-homogeneity at a scale larger than the wavelength and brings a lot of scattering, and the refractive index [RI] is not determined correctly any longer. Also, parameters in the calculation of RI using a refractive ellipsometer are too large to make adequate calculation of RI.

## Results and discussion

By analyzing TEM images of the composites (Figure 2), which are obtained by means of treatment with H_2_S of Zn-siloxane mixtures with poly-(dimethyl)-block-(phenyl)siloxanes, one can see that the nanoparticles appear only after treatment of Zn-siloxanes with H_2_S. Such behavior assumes formation of ZnS nanoparticles, which is favorable at elevated temperatures, migration of Zn ions, and formation of clusters (nanoparticles) of ZnS. It is interesting to note that it does not happen in the case of oxygen, and ZnO nanoparticles do not form in oxygen media at elevated temperatures, so one can assume that sulfides are easier to form in a cluster form than oxides. Particle size was determined basing on TEM images. It was found that the particle size is approximately 3 nm with broad distribuiton around the mean value, and with the increase of ZnS concentration, it increases slightly up to 5 nm - mean value. It can be seen from the optical transmission spectra (Figure 4) that composites with smaller ZnS loading (up to 14 wt.% of Zn) possess very high optical transparency, combined with elevated values of refraction coefficient.

At the same time, for the nanocomposites with smaller ZnS loadings (A11S, A12S), we see no particles both in the TEM images and in the XRD spectra, but these compositions possess higher refractive index than pure polymer matrix. This is due to the fact, from our point of view, that for such small loadings, we have smaller clusters of ZnS, which have an amorphous structure and thus are not visible in the XRD spectra or in the HR-TEM or TEM images.

High optical transparency of samples is due to the fact that there is no Mie scattering for the particles with a size less than *λ*/2, and the Rayleigh scattering intensity is expressed by a well-known formula:

(1) $$I=Io\frac{1+\cos^{2}\theta }{2R^{2}}{\left(\frac{2\pi }{\lambda }\right)}^{4}{\left(\frac{n^{2}-1}{n^{2}+2}\right)}^{2}{\left(\frac{d}{2}\right)}^{6},$$

where *I *is the intensity of the Rayleigh scattering; *I*_*o*_, the initial intensity of light; *θ*, the angle between the falling and the scattering ray; *R*, the distance to the nanoparticle; *d*, the diameter of the nanoparticle; *n*, the refraction coefficient; and *λ*, the wavelength of radiation. *I *depends on the particle size as *d*^6^. Thus, for very small particles with *d *approximately 2 to 3 nm, we obtain almost negligible Rayleigh scattering, thus providing optical transparency of the composites which reached 92% to 93% (Figure 4).

Nevertheless, the composites with higher ZnS loading show much higher scattering, which makes them opaque and shadow-like; thus, for the Zn concentrations of more than 12 wt.%, the compositions become highly scattering. This is probably due to the fact that the agglomeration takes place with the increase of ZnS concentration, and for high ZnS concentrations, the agglomerate size becomes comparable with *λ/*2. Also, for the compositions with high loadings, RI drops down drastically. This reflects, from our point of view, the fact that if the agglomerate of particle size is bigger than approximately 5*λ*, the light starts to refract at the interface between particle agglomerates and the polymer.

The values of the refractive index of nanocomposites up to A12S and from A16S to A18S do fit well using effective media theory prediction, which assumes that

(2) $$\text{R}{\text{I}}_{\text{mix}}=\text{ R}{\text{I}}_{\text{ZnS}}x\;+\;\left(1-x\right)\text{ R}{\text{I}}_{\text{matrix}},$$

where *x *is the volume rate of the component in the mixture, RI_ZnS _= 2.39, and RI_matrix _= 1.55. Thus, for the A12S (1.96 vol.%), calculated according to effective media prediction, RI should have been 1.576, and in fact, it was measured to be 1.57.

We foresee three ranges of agglomerates in particle length scales, which differ in interaction with light: (1) *d *< <*λ*/2 - Rayleigh regime, which has ultra low scattering and high transparency; RI is fitted well with effective media approximation; (2) *d *= *λ*/2 to 5*λ *- Rayleigh scattering + Mie scattering, which has an average value of scattering and still has high transparency; some discrepancies from effective media theory predictions are observed; and (3) *d *> 5*λ *- geometry optics range, which has a lot of scattering; effective media approxiation works. Thus, we believe that for the nanocomposites with Zn of more than 12 wt.% (ZnS of 5.5 vol.%), nanoparticle aggregation in big clusters takes place (we measured XRD for these samples, and still there are no peaks of ZnS, which provides evidence of small nanoparticles but joined in the big aggregates); effective media approximation still works; we obtain composites where big nanoparticle aggregates work as scatterers; and the light is refracted at the polymer-aggregate interface. It is also anticipated that samples A14S and especially A15S are in the Mie scattering range since scattering becomes high, especially for A15S, and the effective media theory gives big discrepancies of RI in comparison with the experimentally observed ones.

Analyzing the dependencies of refractive indices of nanocomposites on ZnS content (Figure 6), it can be seen that the dependence is linear and is fitted with effective media approximation, except for nanocomposites A13S, A14S, and A15S. These nanocomposites are in the Mie scattering mode, according to our assumption, and their refractive indices are well above the values, predicted by effective media theory. The reason of these phenomena is unknown to the authors.

Also, it is necessary to note that the agglomeration of nanoparticles and growth of film inhomogeneity at higher ZnS content reflect the fact that ZnS nanocrystals tend to agglomerate if the concentration is higher than some critical one, minimizing the free surface energy. This provides some insight into the ability to disperse nanoparticles homogeneously in siloxane matrices without agglomeration.

## Conclusion

In the present study, we showed that it is possible to synthesize nanocomposites, containing high amounts of ZnS nanoparticles with a very small size, homogeneously dispersed in a poly-(dimethyl)-block-(phenyl)siloxane [PDMPS] matrix without agglomeration. It becomes possible by the introduction of Zn-siloxane into the PDMPS matrix and following treatment of such co-polymer with H_2_S.

It is interesting to note that only H_2_S treatment of Zn-containing siloxanes leads to the formation of nanoparticles; the same is not true for oxygen, and ZnO is not formed in oxygen atmosphere. Also, the agglomeration of nanoparticles in clusters critically depends on the content of Zn-siloxane in the mixture with PDMPS and ZnS concentration.

The obtained nanocomposites can be used as an optical media with high refraction coefficient since it is possible to increase the refraction index of the composite material from 1.54 for pure polymer matrix up to 1.68 for the A15S nanocomposite. At the same time, composites are optically transparent, with very low scattering, which allows their use as silicon sealants or adhesives in LED production, in solar cells, or as waveguide coatings.

## Competing interests

The authors declare that they have no competing interests.

## Authors' contributions

NS made the synthesis and elemental analysis. DG prepared the films for optical measurements, made the theoretical assumptions, and drafted the manuscript. BZ designed the study and controlled the measurements. ML participated in the TEM and HR-TEM measurements. MK carried out all optical, TEM, and XRD measurements. All authors read and approved the final manuscript.

## Aknowledgments

The authors would like to thank LG Chem Ltd. for the financial support.
